# Structures of synthetic nanobody–SARS-CoV-2 receptor-binding domain complexes reveal distinct sites of interaction

**DOI:** 10.1016/j.jbc.2021.101202

**Published:** 2021-09-16

**Authors:** Javeed Ahmad, Jiansheng Jiang, Lisa F. Boyd, Allison Zeher, Rick Huang, Di Xia, Kannan Natarajan, David H. Margulies

**Affiliations:** 1 Molecular Biology Section, Laboratory of Immune System Biology, National Institute of Allergy and Infectious Diseases, National Institutes of Health, Bethesda, Maryland, USA; 2 Laboratory of Cell Biology, Center for Cancer Research, National Cancer Institute, National Institutes of Health, Bethesda, Maryland, USA

**Keywords:** single-domain antibody (sdAb, nanobody), SARS-CoV-2, surface plasmon resonance (SPR), crystallography, cryo-EM, ACE2, angiotensin-converting enzyme 2, BSA, buried surface area, CCs, correlation coefficients, CDR1, complementarity-determining region 1, CDR2, complementarity-determining region 2, CDR3, complementarity-determining region 3, CTF, contrast transfer function, PDB, Protein Data Bank, RBD, receptor-binding domain, S, spike, S-6P, HexaPro S, SARS-CoV-2, severe acute respiratory syndrome coronavirus 2, SEC, size-exclusion chromatography, SPR, surface plasmon resonance

## Abstract

Combating the worldwide spread of severe acute respiratory syndrome coronavirus 2 (SARS-CoV-2) and the emergence of new variants demands understanding of the structural basis of the interaction of antibodies with the SARS-CoV-2 receptor-binding domain (RBD). Here, we report five X-ray crystal structures of sybodies (synthetic nanobodies) including those of binary and ternary complexes of Sb16–RBD, Sb45–RBD, Sb14–RBD–Sb68, and Sb45–RBD–Sb68, as well as unliganded Sb16. These structures reveal that Sb14, Sb16, and Sb45 bind the RBD at the angiotensin-converting enzyme 2 interface and that the Sb16 interaction is accompanied by a large conformational adjustment of complementarity-determining region 2. In contrast, Sb68 interacts at the periphery of the SARS-CoV-2 RBD–angiotensin-converting enzyme 2 interface. We also determined cryo-EM structures of Sb45 bound to the SARS-CoV-2 spike protein. Superposition of the X-ray structures of sybodies onto the trimeric spike protein cryo-EM map indicates that some sybodies may bind in both “up” and “down” configurations, but others may not. Differences in sybody recognition of several recently identified RBD variants are explained by these structures.

Severe acute respiratory syndrome coronavirus 2 (SARS-CoV-2), a β-coronavirus, is remarkable for its high infectivity, rapid worldwide dissemination, and evolution of highly infectious new variants (1, 2, 3, 4). The virus exploits its trimeric spike (S) glycoprotein to adsorb to the host cell-surface receptor, angiotensin-converting enzyme 2 (ACE2) (5), resulting in proteolytic processing and conformational changes required for membrane fusion and cell entry (6). Understanding the fundamental molecular and cell biology and chemistry of the viral life cycle and the nature of the host immune response offers rational avenues for developing diagnostics, therapeutics, and vaccines (7, 8). Emerging viral variants that exhibit increased infectivity and virulence emphasize the need for continued improvement in immunization and therapeutic approaches. Specifically, B.1.1.7 (United Kingdom), B.1.351 (South Africa), P.1 (Brazil), and other strains demand careful attention (9, 10, 11, 12, 13, 14). Exploring the detailed structures of antiviral antibodies can provide critical understanding of the means to attenuate viral adsorption and entry and prevent or retard ongoing infection and communal spread. An evolving database of X-ray and cryo-EM structures of the SARS-CoV-2 S and receptor-binding domain (RBD) and their interactions with ACE2 or various antibodies contributes to the design of effective antibodies or immunogens (15). Recent studies indicate the value of single-domain antibodies derived from camelids (nanobodies) (16) or camelid-inspired synthetic libraries (sybodies) (17, 18) and the potential effectiveness of multivalent constructs (19). Many properties of nanobodies make them well suited for structural studies and drug development (20). Here, we take advantage of available sequences of five SARS-CoV-2 RBD–directed sybodies: Sb14, Sb15, Sb16, Sb45, and Sb68 (previously designated Sb#14, Sb#15, Sb#16, Sb#45, and Sb#68, respectively (18)). These sybodies effectively inhibit the ACE2–RBD interaction and neutralize viral infectivity (18), and a bispecific construct, consisting of Sb15 linked to Sb68, blocked ACE2 binding and neutralized both pseudotyped and infectious SARS-CoV-2 viruses (18). Here, we describe binding studies and X-ray structures of binary complexes of Sb16–RBD and Sb45–RBD, ternary complexes of Sb14–RBD–Sb68 and Sb45–RBD–Sb68, and unliganded Sb16. In addition, we report cryo-EM structures of Sb45 complexed with trimeric S and evaluate sybody interactions with several mutant RBDs, representative of newly evolving variants.

## Results

### Binding and affinity analysis

**Figure 1:**
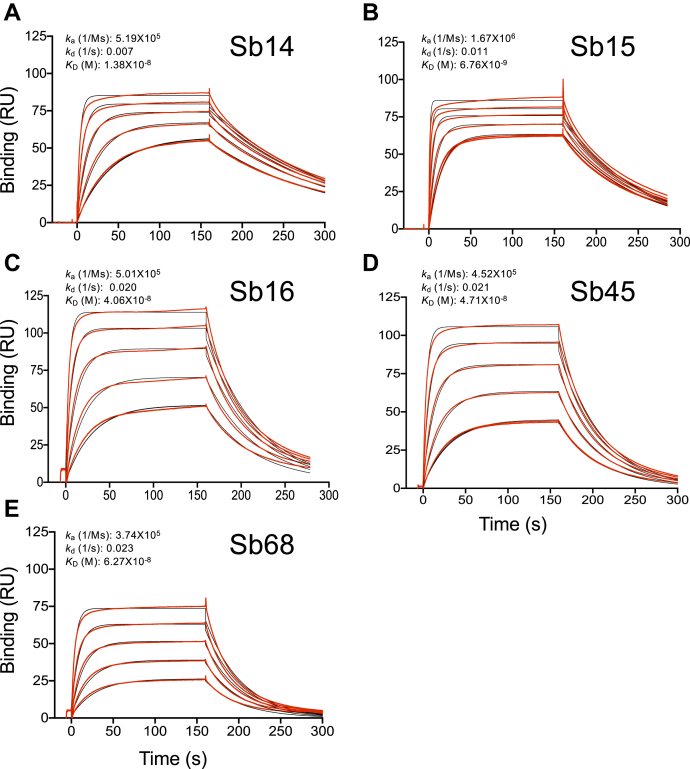
**Sybodies bind the RBD with *K***_**D**_**values in the nanomolar range.** The RBD was coupled to a biosensor chip as described in Experimental procedures. Graded concentrations (31–500 nM) of each of the indicated sybodies were offered to the coupled surface (from t = 0 to t = 160 s) followed by buffer washout and measurement of net binding (in RU). Experimental curves (*red*) were fit by global analysis using Biacore T200 evaluation software 3.1 (Cytiva) (*black*). The *curves* shown are representative of at least two determinations. *A*, *B*, *C*, *D*, and *E* represent data and curve fits for Sb14, Sb15, Sb16, Sb45, and Sb68, respectively. RBD, receptor-binding domain; RU, resonance units.

Sybodies were expressed in *Escherichia coli* and purified *via* metal-affinity chromatography to high purity. These sybodies behaved as monomers by size-exclusion chromatography (SEC) (21) (Fig. S1), and we confirmed their activity in binding to the bacteria-expressed RBD as visualized by SEC (Fig. S1). As determined by surface plasmon resonance (SPR), all five sybodies bind to the immobilized RBD with *K*_D_ values of 6.8 to 62.7 nM, consistent with previous determinations using RBD-YFP or RBD-Fc molecules by related techniques (18) (Fig. 1).

### Structure of sybody–RBD binary and ternary complexes

To gain insight into the precise topology of the interaction of four of these sybodies with the RBD, we determined crystal structures of their complexes: Sb16–RBD and Sb45–RBD, the ternary Sb45–RBD–Sb68 and Sb14–RBD–Sb68, and Sb16 alone. These crystals diffracted X-rays to resolutions from 1.7 to 2.6 Å (Table 1). After molecular replacement, model building, and crystallographic refinement (see Experimental procedures), we obtained structural models that satisfied standard criteria for fitting and geometry (Table 1). Illustrations of the quality of the final models as compared with the electron density maps are shown in Fig. S2.

**Table 1:** X-ray data collection and refinement statistics

Data collection and refinement	Sb16–RBD	Sb45–RBD	Sb14–RBD–Sb68	Sb45–RBD–Sb68	Sb16
PDB ID	7KGK	7KGJ	7MFU	7KLW	7MFV
Data collection					
Space group	P6_5_22	P3_2_21	P2_1_	C222_1_	P6_3_22
Cell dimensions					
*a*, *b*, *c* (Å)^a^	65.64, 65.64, 344.69	62.55, 62.55, 168.82	66.82, 83.05, 92.83	74.50, 102.40, 138.97	68.92, 68.92, 107.17
*α*, *β*, *γ* (°)	90.0, 90.0, 120.0	90.0, 90.0, 120.0	90.0, 106.71, 90.0	90.0, 90.0, 90.0	90.0, 90.0, 120.0
Resolution (Å)	57.34–2.60 (2.69–2.60)	45.59–2.30 (2.38–2.30)	42.17–1.70 (1.76–1.70)	44.12–2.60 (2.69–2.60)	34.46–1.90 (1.97–1.90)
*R*_*sym*_*or R*_*merge*_	0.080 (0.455)	0.101 (0.849)	0.086 (0.765)	0.095 (0.739)	0.074 (1.54)
*I/σ(I)*	18.0 (3.3)	14.9 (3.4)	8.9 (1.7)	13.1 (2.1)	15.2 (0.7)
Completeness (%)	98.8 (99.1)	99.3 (98.3)	98.4 (93.8)	98.8 (98.7)	94.5 (85.0)
Redundancy	10.3 (10.9)	7.9 (8.2)	3.1 (3.1)	7.2 (7.4)	12.4 (12.6)
*R*_*pim*_	0.024 (0.134)	0.038 (0.293)	0.057 (0.510)	0.038 (0.287)	0.022 (1.05)
CC_1/2_	0.999 (0.987)	0.997 (0.919)	0.995 (0.640)	0.998 (0.895)	0.999 (0.526)
Estimated twin fraction	0.0 (none)	0.06 (−h, −k, l)	0.0 (none)	0.0 (none)	0.0 (none)
Refinement					
Resolution (Å)	56.09–2.60 (2.69–2.60)	45.59–2.30 (2.38–2.30)	42.27–1.70 (1.76–1.70)	36.72–2.60 (2.69–2.60)	34.46–1.90 (1.97–1.90)
No. of reflections	13,219 (1185)	17,592 (1687)	105,129 (9993)	16,508 (1627)	11,786 (1025)
*R*_work_/*R*_free_ (%)	25.8/27.7 (36.3/44.2)	18.6/21.6 (24.1/29.8)	18.1/21.5 (27.0/31.6)	20.6/25.5 (29.3/34.5)	23.7/25.1 (35.4/36.7)
No. of atoms	2486	2641	7798	3552	987
Protein	2486	2500	6798	3456	913
Water + ligands	0	141	962 + 38	96	70 + 4
B-factor Wilson/Average	39.3/59.8	26.9/32.9	20.3/26.9	33.9/31.5	31.8/44.9
Protein	59.8	32.8	25.7	31.5	44.8
Water + ligands	0	34.7	34.5 + 40.0	29.5	45.4 + 57.0
RMSDs					
Bond length (Å)	0.002	0.005	0.004	0.003	0.006
Bond angle (°)	0.54	0.74	0.74	0.64	0.96
Ramachandran					
Favored (%)	92.9	97.4	98.3	96.3	94.7
Allowed (%)	7.1	2.6	1.5	3.7	5.3
Outliers (%)	0.0	0.0	0.2	0.0	0.0
MolProbity					
Clashscore (percentile)	5.35 (99th)	4.94 (99th)	1.8 (99th)	5.95 (99th)	6.75 (92nd)

a Values in parentheses are for the highest resolution shell.

**Figure 2:**
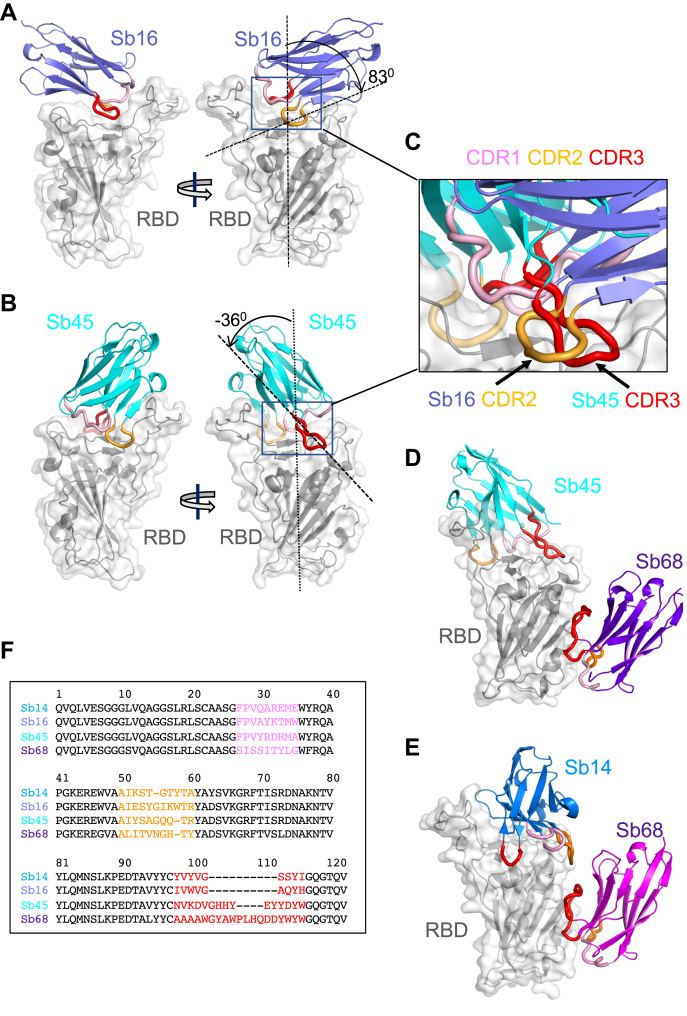
**Overall structures of Sb14, Sb16, Sb45, and Sb68 complexed with the SARS-CoV-2 RBD.***Ribbon* (sybodies) and *ribbon plus surface* (RBD) representations of the complex of (*A*) Sb16 (*slate*) with the RBD (*gray*) (7KGK); (*B*) Sb45 (*cyan*) with the RBD (7KGJ); (*D*) Sb45 and Sb68 (*purple*) with the RBD (7KLW), and (*E*) Sb14 (*blue*) and Sb68 (*magenta*) with the RBD (7MFU). Sb16–RBD and Sb45–RBD superimposed based on the RBD are shown in panel *C*, to highlight CDR loops, which are color-coded as CDR1 (*pink*), CDR2 (*orange*), and CDR3 (*red*). The CDR2 of Sb16 and CDR3 of Sb45 interact similarly with the RBD surface. Panel *F* shows a sequence alignment of the four sybodies. CDR1, complementarity-determining region 1; CDR2, complementarity-determining region 2; CDR3, complementarity-determining region 3; RBD, receptor-binding domain; SARS-CoV-2, severe acute respiratory syndrome coronavirus 2.

**Figure 3:**
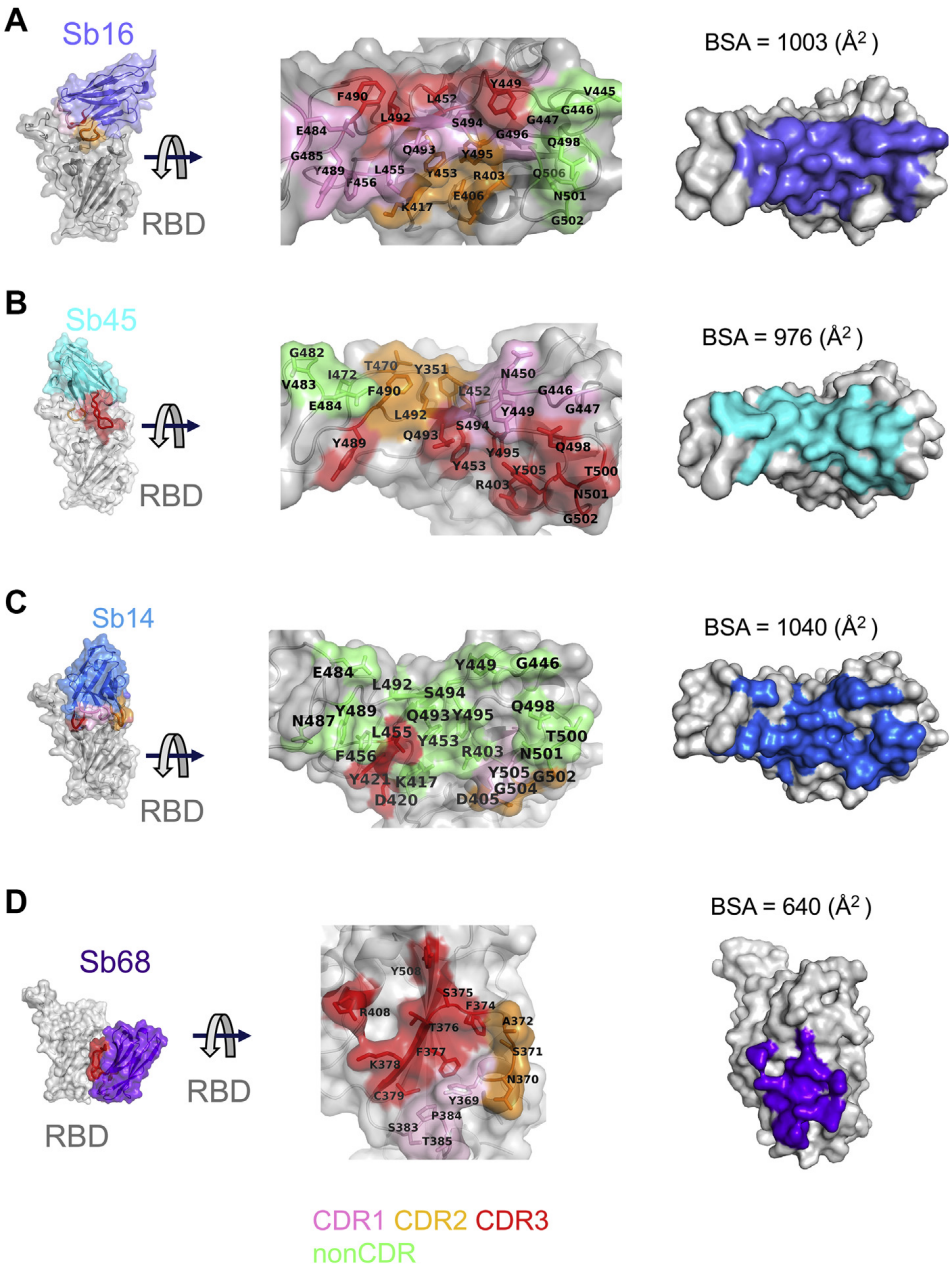
**Interfaces and interactions of sybodies with the RBD.***A*, Sb16–RBD; (*B*) Sb45–RBD; (*C*) Sb14–RBD; and (*D*) Sb68–RBD. (Individual contacting residues are listed in Table S1). CDR1, CDR2, and CDR3 are painted *pink*, *orange*, and *red*, respectively. Additional non-CDR-contacting residues are colored *lime*. On the RBD surface, the epitopic residues that contact the sybodies are colored according to the sybody CDR. CDR1, complementarity-determining region 1; CDR2, complementarity-determining region 2; CDR3, complementarity-determining region 3; RBD, receptor-binding domain.

The structure of the RBD of these complexes (Fig. 2, *A* and *B*) revealed little difference between insect-expressed (22) and our bacteria-expressed and refolded RBDs. Each of the sybodies represents an immunoglobulin variable-type fold (23, 24) consisting of two β-sheets packed as a β-barrel linked by a disulfide bond. The Sb16–RBD complex (Figs. 2*A* and 3*A*) illustrates that complementarity-determining region 2 (CDR2) (residues 50–60) and complementarity-determining region 3 (CDR3) (residues 98–106) bestride the saddle-like region of the ACE2-binding surface of the RBD (see sequence alignment in Fig. 2*F*). Sb16 angulates over the RBD by 83°. However, Sb45 (Figs. 2*B* and 3*B*) straddles the RBD saddle in the opposite orientation, at an angle of −36°, and frames the interface with CDR2 (residues 50–59) and CDR3 (residues 97–111). Complementarity-determining region 1 (CDR1) of both sybodies (residues 27–35) lies between the CDR2 and CDR3 loops. Superposition of the two structures, based on the RBD, emphasizes the diametrically opposite orientation of the two (Fig. 2*C*), revealing that CDR2 of Sb16 and CDR3 of Sb45 recognize the same epitopic regions.

By exploring the conditions using mixtures of two or three sybodies and the RBD, we obtained crystals and solved the structures of ternary complexes consisting of Sb45–RBD–Sb68 at 2.6 Å resolution (Table 1 and Fig. 2*D*) and Sb14–RBD–Sb68 at 1.7 Å resolution (Fig. 2*E*). The refined models revealed that while Sb14 and Sb45 interact with the ACE2 interface of the RBD, Sb68 binds the RBD at a distinct site (Fig. 2, *D* and *E*). In the ternary complex, Sb45 binds in an identical orientation to that observed in the binary Sb45–RBD structure (RMSD of superposition, 0.491 Å for 1981 atoms), but Sb68 addresses a completely different face of the RBD, similar to that bound by Fab of CR3022 on the RBD of SARS-CoV-2 (25) and by V_HH_72 on the RBD of SARS-CoV-1 (26). Of particular interest, whereas Sb45 CDR2 and CDR3 span the RBD saddle as noted above, the distinct contacts of Sb68 to the RBD are through the longer CDR3, with only minor contributions from CDR1 and CDR2. Walter *et al.* (18) visualized similar distinct interactions in cryo-EM maps of two sybodies (Sb15 and Sb68) bound to S protein with local resolution of 6 to 7 Å. Similarly, Sb14, which interacts *via* distinct sybody residues with the RBD at the ACE2 site (see description below), still permits Sb68 to bind to its epitope as seen in the Sb45–RBD–Sb68 structure (Fig. 2*D*).

Scrutiny of the different interfaces provides insights into the distinct ways each sybody exploits its unique CDR residues for interaction with epitopic residues of the RBD (Fig. 3). (Compilation of the contacting residues for each of the four sybodies to the RBD is provided in Table S1). Both Sb16 and Sb45 use longer CDR2 and CDR3 to straddle the RBD, positioning CDR1 residues over the central crest of the saddle (Figs. 2, *A*–*C* and 3, *A* and *B* and Table S1). In addition, several non-CDR residues (Y37, E44, and W47 for Sb16), derived from framework 2 (27), provide additional contacts to the RBD (see Table S1). In contrast with Sb16 and Sb45, Sb14, despite interacting with a large surface area of the RBD, uses both CDR2 and CDR3 on the same side and exploits many non-CDR residues, particularly sheets of β-strand as its binding surface (Fig. 3*C* and Table S1). The interface of Sb68 with the RBD (Fig. 3*D*) is quite different, predominantly exploiting nine CDR3, four CDR2, and one CDR1 residues at the interface (see Table S1).

### Sybodies block ACE2–RBD interaction in discrete ways

**Figure 4:**
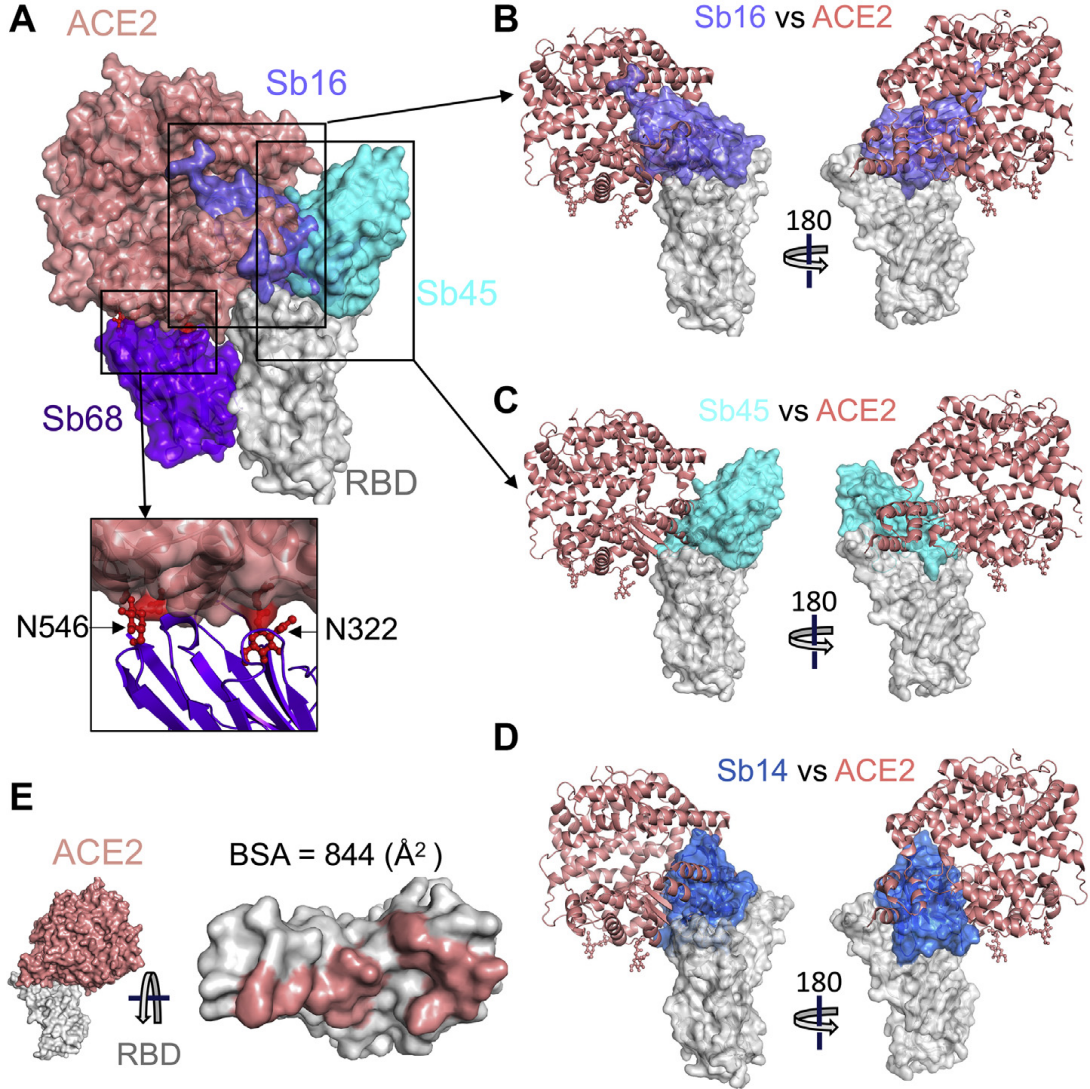
**Sybodies clash with ACE2 in RBD complex structures.***A*, Sb16 (*slate*), Sb45 (*cyan*), Sb14 (*blue*), and Sb68 (*purple*)—RBD complexes were superposed on the ACE2–RBD structure (*salmon*) (6M0J) based on the RBD. Views of Sb16 (*B*), Sb45 (*C*), and Sb14 (*D*) are shown alone as well. Sb14 and Sb16 are buried inside ACE2, Sb45 is partially buried in ACE2, and Sb68 has major clashes with two N-glycan sites (N322 and N546) of ACE2 (inset). *E*, epitopic area (on the RBD) captured by ACE2 (*salmon*) is indicated along with its BSA. ACE2, angiotensin-converting enzyme 2; BSA, buried surface area; RBD, receptor-binding domain.

To evaluate the structural basis for the ability of these four sybodies to block the interaction of the RBD with ACE2, we superposed each of the three sybody–RBD structures onto the ACE2–RBD structure and examined the steric clashes (Fig. 4*A*). Sb16 and Sb45 directly impinge on the ACE2 binding site, offering a structural rationale for their viral neutralization capacity (18). Sb68, which also blocks viral infectivity, binds to the RBD at a site that appears to be noncompetitive for ACE2 binding. The carbohydrate at ACE2 residues N322 and N546 provides an explanation (Fig. 4*A*).

To compare the epitopic areas captured by these sybodies, we evaluated the buried surface area (BSA) interfaces between the RBD and ACE2 or the sybodies. The BSA at the ACE2–RBD, Sb14–RBD, Sb16–RBD, Sb45–RBD, and Sb68–RBD interfaces is 844 Å^2^, 1040 Å^2^, 1003 Å^2^, 976 Å^2^, and 640 Å^2^, respectively (Fig. 3, *A*–*E*). Sb16 and Sb45 capture more surface area than ACE2 or other published nanobody or sybody–RBD complexes (see Table S2). The interface with Sb68 is the smallest (640 Å^2^) (Fig. 3*D*). The total BSA captured by Sb45 and Sb68 in the ternary complex is 1650 (1010 plus 640) Å^2^ (Table S2) and is consistent with the view that a linked bispecific sybody, as described by Walter *et al.* (18), would exert strong avidity effects. Table S2 summarizes these BSA values and those of other nanobody–RBD interactions.

Although Sb68 reveals the smallest BSA with the RBD and binds at a distinct site, it still blocks ACE2 binding. A reasonable explanation for the ability of Sb68 to block the ACE2–RBD interaction arises on inspection of the sites where Sb68, bound to the RBD, might clash with ACE2. Scrutiny of a superposition of Sb68–RBD with ACE2–RBD reveals several areas of steric interference. Sb68 loops 40 to 44 clash with amino acid side chains of ACE2 (residues 318–320 and 548–552), loops 61 to 64 with ACE2 N322 carbohydrate, and loops 87 to 89 (a 3_10_ helix) with ACE2 N546 carbohydrate as well as residues 313 and 316 to 218 (Fig. 4*A*). ACE2 used in the crystallographic visualization of ACE2–RBD (28) was expressed in *Trichoplusia ni* insect cells, which produce biantennary N-glycans terminating with N-acetylglucosamine residues (29, 30). Electron density was observed only for the proximal N-glycans at residues N322 and N546, but larger, complex, nonsialylated, and biantennary carbohydrates have been detected in glycoproteomic analysis of ACE2 in mammalian cells (31). These carbohydrates are highly flexible, adding greater than 1500 Da at each position and are larger than the single carbohydrate residues visualized in the crystal structure. In addition, molecular dynamics simulations of ACE2–RBD implicated the direct interaction of carbohydrate with the RBD (32). Thus, the ability of Sb68 to impinge on ACE2 interaction with the RBD likely involves the steric clash of the N322- and N546-linked glycans.

We also obtained a 1.9 Å structure of free Sb16 (Fig. S3). Remarkably, CDR2 of Sb16 shows Y54 in starkly different positions in the unliganded structure as compared with the complex: the Cα carbon is displaced by 6.0 Å, while the Oη oxygen of Y54 is 15.2 Å distant, indicative of dynamic flexibility.

### Analysis of cryo-EM maps of Sb45–trimeric S complexes

**Figure 5:**
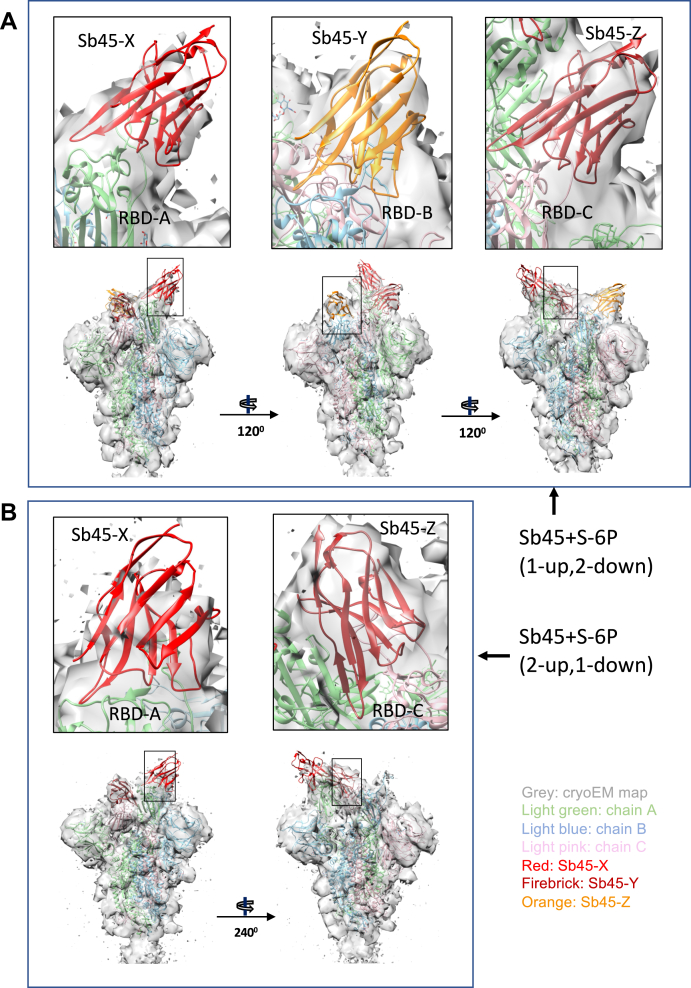
**X-ray model of sybody superposed on cryo-EM structures of SB45–S-6P.***A*, model of Sb45+S-6P (1-up, 2-down) is fitted to the map with Sb45-X bound to RBD-A (up); Sb45-Y to RBD-B (down), and Sb45-Z to RBD-C (down). CCs (Sb45-X/Sb45-Y/Sb45-Z) are 0.52/0.49/0.57 respectively. *B*, model of Sb45+S-6P (2-up, 1-down) is fitted to the map with Sb45-X bound to RBD-A (up) and Sb45-Z bound to RBD-C (down), and CCs (Sb45-X/Sb45-Z) are 0.47/0.70, respectively. CCs, correlation coefficients; RBD, receptor-binding domain.

To gain further insight into the interaction of Sb45 with the full S protein, we prepared complexes of Sb45 with HexaPro S (S-6P), a stable S variant containing six beneficial proline substitutions (33) and acquired cryo-EM images as described in Experimental procedures. All image processing, 2D class, 3D reconstruction, and map refinements were performed with cryoSPARC (34, 35, 36, 37), model fitting with Chimera, (38) and refinement with PHENIX (39). We identified two conformations of S-6P with the RBD in either a 1-up, 2-down (7N0G/EMD-24105) or 2-up, 1-down (7N0H/EMD-24106) position as determined by 3D classification (3D *ab initio* reconstruction) (Fig. S4). We have built in additional loops of the N-terminal domain (NTD) and glycans based on the models of 6XKL, 7KGJ, and 7B62. We used unsharpened maps for model refinement. The overall correlation coefficients (CCs) (mask/volume/peaks) of models for 7N0G and 7N0H are 0.84/0.84/0.77 and 0.83/0.83/0.77, respectively. The model quality is shown in Table 2. There are three Sb45s binding to the 1-up, 2-down form of S-6P (7N0G/EMD-24105); one binds the up position of the RBD and two bind the down position of the RBD (Fig. 5*A*), with CC values of 0.51, 0.49, and 0.58, respectively (Fig. S5, *A*–*C*). Only two Sb45s bind to the 2-up, 1-down form of S-6P (7N0H/EMD-24106), with one on the up position of the RBD and the other on the down position of the RBD (Fig. 5*B*), with CC values of 0.51 and 0.71, respectively (Fig. S5, *D* and *E*). It seems that Sb45 can bind all the down positions of the RBD. In particular, Sb45-Z binds well to RBD-C with higher CC values (Fig. S5, *C* and *E*), with additional contacts to the neighboring (up position) RBD-A (Fig. 5*A*). These variations in saturation of the available conformations by Sb45 reflect the mobility of the RBD. Notably, the interfaces between Sb45 and the RBD of S-6P are the same as those in the crystal structure (7KGJ) (Fig. 2). Moreover, the RBDs are compressed down toward the center of S, approximately 2 to 4 Å as compared with uncomplexed S-6P (6XKL).

**Table 2:** Cryo-EM data collection, refinement, and validation statistics

Data collection, refinement, and validation	Sb45+S-6P (1-up, 2-down)	Sb45+S-6P (2-up, 1-down)
EMDB ID	EMD-24105	EMD-24106
PDB ID	7N0G	7N0H
Data collection and processing		
Magnification	130,000	130,000
Voltage (kV)	300	300
Electron exposure (e^−^/Å)	56	56
Defocus range (μm)	−0.7 to −2.0	−0.7 to −2.0
Pixel size (Å/pixel)	0.526 (1.052 binned)	0.526 (1.052 binned)
Raw micrographs (No.)	9725	9725
Extract particles (No.)	1,447,993	1,447,993
Selected 2D particles (No.)	662,994	662,994
Refined particles (No.)	417,460	417,460
Particles for final map (No.)	214,171	60,062
Symmetry imposed	C1	C1
Map resolution (Å)	3.02	3.34
FSC threshold	0.143	0.143
Refinement		
Initial model used	6XKL, 7KGJ	6XKL, 7KGJ
Model composition		
Atoms	29,062	27,974
Residues	3592	3469
Ligands (NAG)	73	64
Overall B-factor (Å^2^)		
Protein (min/max/mean)	36.8/589.6/157.0	24.2/485.3/157.0
Ligands (min/max/mean)	55.3/340.1/129.9	51.8/358.8/144.5
RMSDs		
Bond length (Å)	0.003	0.005
Bond angle (°)	0.548	0.972
CC (mask/volume/peaks)	0.84/0.84/0.77	0.83/0.83/0.77
Validation		
MolProbity score	1.62	1.71
Clashscore	7.71	8.26
Poor rotamers	0.00	0.00

We did not use sharpened maps, and autosharpening by PHENIX did not improve the CC, so we kept the unsharpened maps for the refinement.

### Superposition of sybodies on trimeric S protein models

To gain additional insight into the structural consequences of the interactions of each of these sybodies with a trimeric S protein, we superposed each of the individual sybody–RBD complexes on S-6P of our cryo-EM structures (7N0G and 7N0H) (Fig. S6). Sb16 and Sb45 may dock on all three RBDs in the trimeric S in any of the four configurations, without any apparent clash (Fig. S6, *A* and *B*). Sb14, however, reveals clashes when the Sb14–RBD complex is superposed on trimeric S in any down position (Fig. S6*E*). Sb68 could not be superposed without clashes to any RBD of the 3-down or to the 1-up, 2-down positions. The only permissible superpositions were to two in the 2-up, 1-down and to all three in the 3-up positions (Fig. S6*F*). For paired sybodies, Sb16 and Sb68 (Fig. S6*B*), or Sb45 and Sb68 (Fig. S6*D*), superposition was possible without clashes, with two or more RBDs in the up conformation. We also observed direct interactions of Sb14 and Sb68 in the Sb14–RBD–Sb68 X-ray crystal structure. Walter *et al.* (18) suggested that a covalent bispecific Sb15–Sb68 reagent could bind S in both the 2-up and 3-up configurations, based on cryo-EM maps of complexes of S with Sb15 and Sb68. It appears that Sb16 binds to S in an orientation similar to but in detail distinct from that of Sb15. This analysis demonstrates an advantage of the small size of sybodies or nanobodies in accessing epitopic regions of S (Fig. S7*C*).

### Binding to RBD mutants

**Figure 6:**
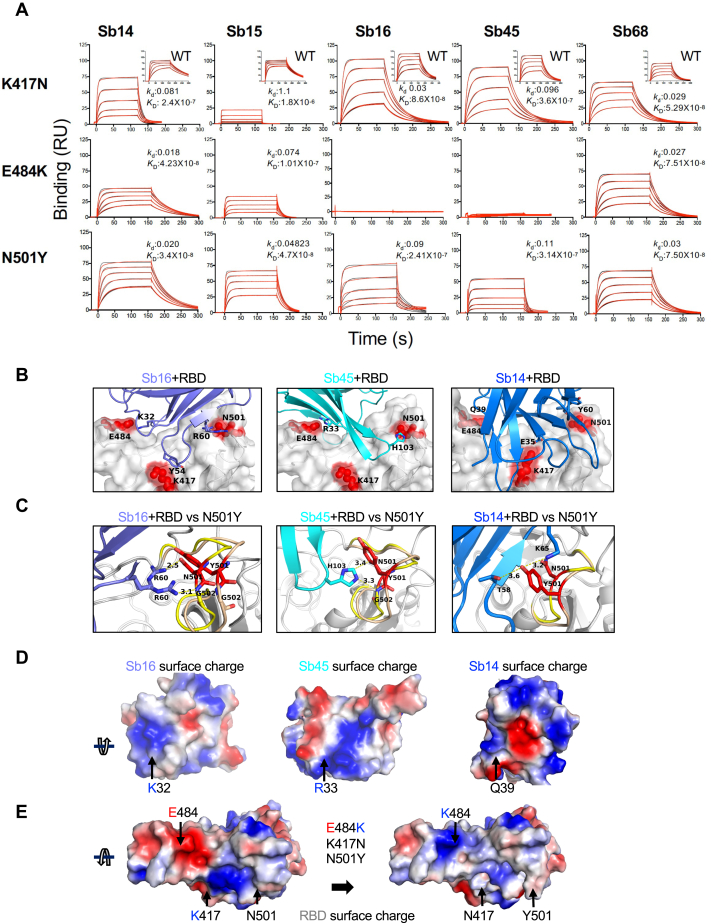
**RBD mutations affect sybody binding.***A*, SPR binding of each of the indicated sybodies (*across top*) to each of the individual RBD mutants. *Inset* shows binding of sybodies to the WT RBD (from Fig. 1). Experimental tracings are shown in *red*, curve fits in *black*, and *k*_d_ (s^−1^) and *K*_D_ (M) values as determined from global fitting with BIAeval 2.0 are provided in each panel. *B*, location of contacts of Sb16, Sb45, and Sb14 is shown. E484, K417, and N501 of the RBD (WT) interact with K32, Y54, and R60 of Sb16, respectively; E484 and N501 of the RBD (WT) interact with R33 and H103 of Sb45, respectively; and E484, K417, and N501 of the RBD (WT) interact with Q39, E35, and Y60 of Sb14, respectively. *C*, comparison of complex structures with minimized models involving the N501Y mutation. *In silico* mutagenesis of N501Y was performed using 7KGK (Sb16+RBD), 7KGJ (Sb45+RBD), and 7MFU (Sb14+RBD+Sb68). After amino acid substitution in Coot, local energy minimization (within 15–20 Å of the mutant residue) was performed through three rounds in PHENIX. For the Sb16–RBD complex, when N501 is mutated to Y501, the loop (496–506, from *yellow* to *wheat*) extends about 2.4 Å, but R60 (revealing a double conformation) still forms hydrogen bonds with the Y501 loop; for the Sb45–RBD complex, when N501 is mutated to Y501, the loop (496–506, from *yellow* to *wheat*) extends about 1.0 Å, but H103 of Sb45 would still interact with Y501; for the Sb14–RBD complex, when N501 is mutated to Y501, the loop (496–506, from *yellow* to *wheat*) is extended about 2.0 Å, but T58 and K65 still form hydrogen bonds with Y501. *D*, the surface charge of Sb16; K32 forms a hydrogen bond with E484 of the RBD with the opposite charge; the surface charge of Sb45, R33 forms a hydrogen bond with E484 of the RBD with the opposite charge; the surface charge of Sb14, Q39 (a neutral residue) interacts with E484 of the RBD. *E*, surface charge of the WT RBD and surface charge of the RBD with the three mutations (E484, K417N, and N501Y). When E484 is mutated to K484, the surface charge is changed from negative to positive. Therefore, the hydrogen bonds are broken, pushing Sb16 and Sb45 out of contact, whereas because Q39 of Sb14 is not a charged residue, it still may interact with K484 of the mutated RBD. RBD, receptor-binding domain; SPR, surface plasmon resonance.

The major circulating variants, specifically B.1.1.7 (United Kingdom), B.1.351 (South Africa), and P.1 (Brazil), contain mutations in the RBD that lead to increased binding affinity to ACE2 and have the potential to reduce vaccine efficacy (4, 14, 40, 41, 42). Specifically, in addition to other mutations throughout the S protein and viral genome, all three harbor N501Y. B.1.351 and P.1 also have the E484K substitution, as well as the substitution of K417 (to N for B.1.351 and to T for P.1). To assess the effect that substitution at each of these positions exerts on reactivity with Sb14, Sb15, Sb16, Sb45, and Sb68, we engineered individual mutations in the RBD and tested them by SPR (Fig. 6*A*). In general, the five sybodies that interact with the parental (designated WT) RBD with *K*_D_ values of 6.8 × 10^−9^ (for Sb15) to 6.3 × 10^−8^ M (for Sb68) (Fig. 1) showed different patterns of binding to the K417N, E484K, and N501Y mutants. Sb68 bound each with similar affinity, consistent with its epitope lying outside of the ACE2 binding site on the RBD, whereas each of the others revealed a distinct pattern. Sb14 binding was most affected by K417N. Sb15 bound both K417N and E484K less efficiently than N501Y. Sb16, largely unaffected in binding to K417N, showed decreased recognition of N501Y and failed to interact detectably with E484K. Similar to Sb16, Sb45 also failed to bind E484K and showed decreased recognition of K417N and N501Y as compared with WT. To understand the structural basis of these differences in recognition of the different RBD mutants, we generated models based on the sybody–RBD structures (Fig. 6, *B*–*E*). For Sb16, Sb45, and Sb14, interaction with the N501Y mutant resulted in displacement of loops 496 to 506 by 2.0 Å, 1.0 Å, and 1.5 Å, respectively. Nevertheless, R60 of Sb16 and H103 of Sb45 maintained contact with N501Y. This suggests that N501Y mutation would not escape recognition by these sybodies. Other cryo-EM studies indicate modest effects of the N501Y substitution on binding to different antibodies (43). In contrast to the effects of N501Y, E484K revealed major incompatibilities because of charge repulsion in the interaction with Sb16 *via* K32 and Sb45 *via* R33 (Fig. 6, *D* and *E*).

## Discussion

Our studies of the X-ray structures of Sb16 alone, Sb16–RBD, Sb45–RBD, and the ternary Sb14–RBD–Sb68 and Sb45–RBD–Sb68 complexes and the cryo-EM structures of Sb45–S provide critical details describing the basis of the inhibition of S binding to the cell-surface ACE2 receptor and the resulting block of viral infectivity. Sybodies and nanobodies, by virtue of their single-domain structure and ability to be expressed in *E. coli* systems, as noted by others (17, 19), offer advantages over Fab. Our X-ray structures (at resolutions 1.7–2.6 Å) are complemented by the recent preliminary report of cryo-EM-based models of Sb15 and Sb68 bound to S (18). Although those cryo-EM structures show overall good resolution (around 3 Å), local resolution was significantly worse (6–7 Å). Our new structures increase the understanding of the details of sybody interactions with the RBD, and in the case of Sb16, emphasize the dynamic role played by CDRs in adjusting to epitopic surfaces. Barnes *et al.* (44) categorized a host of anti-S and anti-RBD Fabs into four classes (1 to 4) based on the location of the footprint and whether the Fab has access to either the up only or up and down configuration of the RBD in the context of the full trimer (Fig. S7*A*). By superposition (Fig. S7*A*), Sb14 clearly belongs to class 1 because it completely covers the light chain of B38 Fab (7BZ5). Sb16 partially clashes with B38, but it primarily overlaps with the heavy chain of COVA2-39 (7JMP). It can bind both to up and down positions of the RBD in S (Fig. S6), indicating that it belongs to class 2 (Fig. S7*A*). Sb45 clashes effectively with the heavy chain of COVA2-39, and our cryo-EM structures (7N0G and 7N0H) indicate that Sb45 can bind to both up and down forms of S-6P (Fig. 5). Thus, Sb45 qualifies as class 2 (Fig. S7*A*). By contrast, Sb68 competes mostly with the CR3022 heavy chain (6W41), V_HH_72 (6WAQ) (26) and V_HH_-U (7KN5) (45) placing it in class 4. However, unlike the other class 4 antibodies, Sb68 competes presumably because of its spatial orientation. Overall, our structural studies not only define the Sb14, Sb16, Sb45, and Sb68 epitopes at high resolution but also reveal that these sybodies capture a rather large epitopic area (Table S2), suggesting that a judicious choice of several sybodies or nanobodies has the potential to effectively saturate the available RBD surface. Based on the design of the sybody libraries, Walter *et al.* (18) considered Sb14 and Sb16 as “concave,” Sb45 as “loop,” and Sb68 as “convex.” The X-ray structures indicate that although Sb14 and Sb16 are of the same group, Sb14 interacts with the RBD primarily through non-CDR residues (Fig. 3), whereas Sb16 binds through CDR1, CDR2, and CDR3 (Fig. 3). Sb45, which is oriented differently at the ACE2–RBD interface, exploits all three CDRs as well as non-CDR residues (Fig. 3). Sb68, on the other hand, exploits its long CDR3 to bind at its distinct site. Thus, it is possible that convex sybodies may offer an opportunity for identifying distinct epitopes.

The significance of the ternary structures of Sb45–RBD–Sb68 (7KLW) and Sb14–RBD–Sb68 (7MFU) is shown in a recent article (45). Koenig *et al.* (45) determined a ternary nanobody structure of V_HH_-E–RBD–V_HH_-U (7KN5) that illustrates the binding to two distinct epitopic sites. The ternary structure may also be considered as illustrative of the potential behavior of a bispecific construct linking two nanobodies. The bivalent or multivalent binding by the antibody or nanobody would be expected to increase neutralization potential (19, 45, 46, 47). Superposition of Sb14–RBD–Sb68 or Sb45–RBD–Sb68 on V_HH_-E–RBD–V_HH_-U indicates that Sb14, Sb45, and V_HH_-E represent class 1 and class 2 in recognizing the epitopic region but do so in somewhat different orientations (Fig. S7*B*). Sb45 exploits its two lengthy CDR2 and CDR3 loops that ride along both sides of the RBD surface, and Sb14 uses both CDR2 and CDR3 on the same side close to Sb68, while V_HH_-E uses a long CDR3 loop engaging one side of the RBD surface. Furthermore, Sb14 and Sb68 in Sb14–RBD–Sb68 (7MFU) show contacts (Y57-E44, G55-E44, and T54-H108) between two specific sybodies on the RBD surface (Fig. S7*C*), which emphasizes the potential benefit of using complementary, bivalent, or multivalent antibodies/nanobodies against the virus.

Recently, several SARS-CoV-2 S variants have been isolated and characterized with respect to their infectivity and severity of disease. The UK-SARS-CoV-2 variant has multiple substitutions including N501Y in the RBD (1). The mutation of E484K leads to the repulsion of charged residues of the antibody/nanobody/sybody (Fig. 6). To accommodate such a mutation, the complementary charged residues of the antibody/nanobody/sybody should also reverse their charge. Alternatively, using another antibody/nanobody/sybody with opposite charge could capture such an escape mutation. Indeed, knowledge of the location of common or recurrent escape mutations and their potential resistance to the antibody/nanobody/sybody would provide a rational basis for either sequential or simultaneous use of reagents with complementary specificity. Thus, precise mapping of anti-RBD antibody, nanobody, and sybody epitopes, especially for those that are developed for clinical trials, has implications not only for mechanistic understanding of the interactions of the RBD with ACE2 but also for evaluating the potential susceptibility of newly arising viral variants to currently administered vaccines and antibodies.

## Experimental procedures

### Subcloning, expression, and purification of RBD, S, and sybody proteins

The sequences encoding the RBD of the SARS-CoV-2 S protein (amino acids 333–529) were subcloned into pET21b(+) (Novagen) *via Nde*I and *Eco*RI restriction sites, using pcDNA3–SARS-CoV-2–RBD–8his (Addgene #145145, (48)) as the template. The primers used were the forward primer, 5′-TGCAGTCATATGAATCTTTGTCCGTTCGGTGAG, and the reverse primer, 5′-TGCAGTGAATTCTCACCCTTTTTGGGCCCACAAACT. The RBD was expressed as inclusion bodies in *E. coli* strain BL21(DE3) (Novagen). Expression and isolation of inclusion bodies, denaturation, and reduction were done in 6 M guanidine hydrochloride and 0.1 mM DTT as described elsewhere (49). Briefly, refolding was carried out in a refolding buffer supplemented with oxidized and reduced glutathione and arginine for 3 days at 4 °C followed by dialysis against Hepes buffer (25 mM Hepes, pH 7.3, and 150 mM NaCl). Concentrated and filtered protein was analyzed using SEC on a Superdex 200 10/300GL column (GE Healthcare) equilibrated with Hepes buffer. The peak corresponding to 24-kDa (monomeric) protein was collected, concentrated, and further purified by ion-exchange chromatography on Mono-Q (Cytiva). Mutant RBDs were generated by site-directed mutagenesis, performed with the QuikChange Lightning Multi Site-Directed Mutagenesis kit (Agilent). All mutants were sequenced through GENEWIZ, and protein expression, refolding, and purification were carried out as described above.

Plasmids pSbinit encoding sybodies Sb14, Sb15, Sb16, Sb45, and Sb68 (Addgene #153522, #153523, #153524, #153526, and #153527, respectively) were originally reported by Walter *et al.* (18) and generously made available. All plasmids were verified by DNA sequencing. Purification of the recombinant proteins from the periplasm of *E. coli* MC1061 was based on a protocol described elsewhere (21). Briefly, *E. coli* MC1061, transformed with a sybody-encoding plasmid, was grown in Terrific Broth medium (Gibco) supplemented with 25 μg/ml chloramphenicol, at 37 °C with shaking at 160 rpm for 2 h. The temperature was then decreased to 22 °C until *A*_600_ reached 0.5. Protein expression was induced by the addition of L-(+)-arabinose (Sigma) to a final concentration of 0.02% (w/v) and expression continued overnight at 22 °C and 160 rpm. The next day, cells were collected by centrifugation at 2000*g* for 15 min. The cell pellet was then washed twice in PBS and resuspended in the periplasmic extraction buffer (50 mM Tris/HCl, pH 8.0, 0.5 mM EDTA, 0.5 μg/ml lysozyme, and 20% w/v sucrose (Sigma)) at 4 °C for 30 min followed by the addition of TBS (pH 8.0) and 1 mM MgCl_2_. The cells were then centrifuged at 10,000 rpm (Fiberlite F21-8 x 507 Fixed-Angle Rotor) for 30 min. After transfer of the supernatant to a fresh tube, imidazole was added to a final concentration of 10 mM. Ni-NTA resin (QIAGEN) equilibrated with TBS was added to the supernatant and incubated for 1 h at room temperature with mild agitation. The resin was collected and washed three times with the buffer supplemented with 30 mM imidazole, and sybody proteins were eluted with 300 mM imidazole in TBS.

Plasmid encoding S HexaPro (designated “S” throughout) was procured from Addgene (#154754) (33) and transfected into Expi293F cells (Thermo Fisher Scientific) using manufacturer’s protocol. Briefly, Expi293F cells were seeded to a final density of 2.5 to 3 × 10^6^ viable cells/ml and grown overnight at 37 °C in Expi293 Expression Medium (Gibco). The following day, cell viability was determined, and cell density was adjusted to 3 × 10^6^ viable cells/ml with fresh, prewarmed Expi293 Expression Medium. Transfection was then carried out as per manufacturer’s instructions using 1 μg/ml plasmid DNA. Cultures were grown for 6 days after transfection, and the supernatant was collected, filtered through a 0.22-μm filter, and passed over Ni-NTA resin for affinity purification. Further purification was accomplished by SEC using a Superose 6 10/300GL column (Cytiva) in a buffer consisting of 2 mM Tris, pH 8.0, and 200 mM NaCl. The purification of sybodies, RBDs, and S-6P is shown in Fig. S8.

### Preparative and analytical SEC

Sybodies purified by Ni-NTA affinity chromatography were concentrated using Amicon 10K MWCO concentrators and purified on a Sepax SRT-10C SEC100 column at a flow rate of 1 ml/min. Monomeric sybodies elute at a retention volume of 11 to 12.5 ml from the Sepax SRT-10C SEC100 column. Monomeric peak fractions were collected and analyzed by SDS-PAGE. Analytical SEC of the RBD–sybody complexes was performed on a Shodex KW-802.5 column at a flow rate of 0.75 ml/min in TBS buffer (pH 8.0). (The interaction of individual sybodies with the column matrix is a well-documented phenomenon (21)).

### SPR

SPR experiments were performed on a Biacore T200 system (Cytiva) at 25 °C in 10 mM Hepes, pH 7.2, 150 mM NaCl, 3 mM EDTA, and 0.05% Tween-20. The RBD was immobilized on a series S CM5 sensor chip (Cytiva) by amine (N-hydroxysuccinimide/1-ethyl-3-(3-dimethylaminopropyl) carbodiimide) coupling to flow cells. For background subtraction, a reference cell was mock coupled. Binding and kinetic studies were performed multiple times for each sybody. Graded and increasing concentrations of SB16, SB45, and SB68 were injected over the RBD-immobilized surface at a flow rate of 30 μl/min, with an association time of 120 s and dissociation time of 2000 s. Binding data were analyzed by surface site affinity distribution analysis with EVILFIT (50, 51) and were consistent with fits to the Langmuir binding equation for a 1:1 interaction model using Biacore T200 Evaluation Software, v3.1.

### Thermal stability

Thermal melt analysis of the recombinant proteins was performed in triplicate in 96-well plates in a QuantStudio 7 Flex real-time PCR machine (Applied Biosystems). Each well contained 2- to 4-mg protein in the buffer (25 mM Tris, pH 8, and 150 mM NaCl) and 5× SYPRO Orange (Invitrogen, stock 5000×) in a total volume of 20 ml. After an initial 2-min hold at 25 °C, the plate was heated to 99 °C at a rate of 0.05 °C/s. The data were analyzed with Protein Thermal Shift Software, v1.3 (Invitrogen), to obtain T_m_ values for the RBD, S, Sb14, Sb15, Sb16, Sb45, and Sb68 (Fig. S9).

### Crystallization, data collection, structure determination, and crystallographic refinement

Purified sybodies (Sb14, Sb15, Sb16, Sb45, and Sb68) and RBDs were mixed in an approximate 1:1 M ratio to a final concentration of 8 mg/ml. The protein mixtures were incubated on ice for 1 h before screening. Initial screening for crystals was carried out using the hanging-drop vapor-diffusion method using the mosquito robotic system (sptlabtech.com). The crystals of SB16–RBD and SB45–RBD complexes and Sb16 alone were observed within 1 week using Protein Complex (QIAGEN) and Wizard Classic 4 (Rigaku). The conditions for Sb16–RBD were either 0.1 M Hepes, pH 7.0, 15% PEG 20000, or 0.1 M Hepes, pH 7.0, and 18% PEG 12000, and for Sb45–RBD, were 18% PEG 12000 and 12% PEG 8000, 0.1 M Hepes, pH 7.5, and 0.2 M NaCl. The crystallization condition for Sb14–RBD–Sb68 was 12% PEG 8000, 0.1 M Mops, pH 7.5, and 0.1 M magnesium acetate. Sb16 alone crystallized in 20% PEG 4000, 0.1 M MES, pH 6.0, and 0.2 M LiSO_4_. We also screened mixtures of two or three sybodies with the RBD. The crystals of Sb45–RBD–Sb68 were obtained after 1 month after mixing the three proteins in an equimolar ratio in 10% PEG 8000 and 0.1 M sodium cacodylate, pH 6.0.

Crystals of protein complexes were optimized with slight adjustments of the concentration of PEG components. The crystals were cryoprotected in the mother liquor containing 5% ethylene glycol and 5% glycerol and flash-frozen in liquid nitrogen for data collection. Diffraction data were collected at the Southeast Regional Collaborative Access Team beamline 22ID-D at the Advanced Photon Source, Argonne National Laboratory, and the data were processed with XDS (52). Multiple datasets were collected for the protein complexes from 2.3 to 3.2 Å resolution. The initial model of Sb16 and Sb45 for the molecular replacement search was built by the MMM server (manaslu.fiserlab.org/MMM (53)), using the heavy-chain V domain and the RBD of the Fab B38–RBD complex (Protein Data Bank [PDB] ID: 7BZ5) (22). The initial model of Sb68 for molecular replacement was built based on the V_H_ domain of 7BZ5. Molecular replacement solutions were found using Phaser (39, 54). Subsequent refinements were carried out using PHENIX (55). CDR loops were manually rebuilt by fitting to the electron density maps with Coot (56). In particular, Sb68 CDR loops were deleted before refinement and built in manually based on electron density maps. Illustrations and calculations of superpositioned models were prepared in PyMOL (The PyMOL Molecular Graphics System, version 2.4.0, Schrödinger, Inc). Calculation of hinge relationships of domains was accomplished with HINGE (https://niaidsis.niaid.nih.gov), provided courtesy of Peter Sun, National Institute of Allergy and Infectious Diseases. Hinge calculates an ellipsoid (defined by axes *a*, *b*, and *c*) for the indicated domains and reports the angle between the long axes of the adjacent domains as the hinge angle. BSA calculations were performed with PISA (https://www.ebi.ac.uk/pdbe/pisa/). The final structures for the RBD–SB16 and RBD–SB45 complexes showed *R*_work_/*R*_free_ (%) of 25.4/27.7 and 18.6/21.6, respectively, and for SB16 alone, *R*_work_/*R*_free_ of 23.7/25.1. Data collection and structure refinement statistics are provided in Table 1.

### Cryo-EM sample preparation and data collection

Freshly purified S-6P was incubated with Sb45 in a 1:3 M ratio and repurified by SEC. Negative stain screening was accomplished with a Tecnai 12 120-keV microscope (Thermo Fisher). We screened several sybody–S complexes for good negative staining images, and complexes of Sb45–S gave the best data. The protein complexes were concentrated to 0.7 to 1 mg/ml, and 3 μl of the sample was applied onto holey-carbon cryo-EM grids (Cu R1.2/1.3, 300 mesh, Quantifoil), which had been glow-discharged for 60 s, blotted for 3 s, and plunge-frozen into liquid ethane with a Vitrobot (Thermo Fisher Scientific) at 4 °C and 95% humidity. Cryo-EM data in selected grid regions were collected on a Titan Krios 300-keV microscope (Thermo Fisher). Images were acquired automatically with SerialEM (57) on a BioQuantum-K2 summit detector (Gatan), with a 20 eV energy filter slit in super-resolution mode at 130× nominal magnification (1.052 Å binned pixel size) and a defocus range from −0.7 to −2.0 μm. An exposure time of 8 s at 0.2 s per frame was recorded with a total exposure of about 56 electrons/Å^2^. Two raw datasets were collected on two frozen grids: one with 1780 micrographs and one with 7945 micrographs.

### Image processing and structure solution

All image processing, 2D class, 3D reconstruction, and map refinements were performed with cryoSPARC, v3.1 and v3.2 (34, 35, 36, 37). A total of 9725 micrographs were imported into cryoSPARC. After “patch motion correction” and “patch contrast transfer function (CTF) estimation,” the number of micrographs was reduced to 9703. The micrographs were inspected by “curate exposures,” in which outliers of defocus range, defective micrographs, and those with a low-resolution estimation of the CTF fit (>5 Å) were discarded, resulting in 9237 micrographs. “Blob picker” was used with the particle diameter between 128 and 256 Å for picking particles. After “inspect particles” with normalized correlation coefficient of 0.28 and “power threshold” between 500 and 1000 (which removed ice and aggregates), the number of particles was 1,876,941. To determine the “box size,” we performed several trials indicating that the box size should be larger than 336 pixels and finally used a box size of 400 pixels and extracted 1,433,963 particles. After “2D classification” (100 classes), 18 2D classes were selected, retaining 662,994 particles. The particles were submitted to a series of “*Ab initio* 3D reconstruction” classification and divided into two or four subgroups. After removing the particles of unrecognized or “defective” shape, a total of 417,460 particles with shape resembling S remained. These particles were subjected to “homogeneity refinement,” followed by “CTF global and local refinement” and “nonuniform refinement.” No symmetry was imposed aside from C1 during the map refinements. The map after refinement could reach 2.84 Å resolution by the gold-standard FSC estimation with a 0.143 cut-off criterion. We then further identified the two conformations of S-6P as previously described (33). One subclass of 214,171 particles revealed the conformation of “1-up, 2-down” of the RBD (Fig. S4*C*), and one subclass of 61,062 particles showed the conformation of “2-up, 1-down” (Fig. S4*C*). The maps of “1-up, 2-down” and “2-up, 1-down” were refined at 3.02 Å and 3.34 Å resolution, respectively. The local resolution plots for each map are shown in Fig. S4, *D* and *E*. The maps are deposited in the Electron Microscopy Data Bank as EMD-24105 and EMD-24106.

An initial model for S-6P was generated using PDB ID: 6XKL and was fitted as a rigid body into the map using Chimera (38) followed by PyMOL. The Sb45–RBD (7KGJ) crystal structure was superimposed onto the S-6P model in PyMOL. We used real-space refinement in PHENIX (39) including rigid-body refinement. The model was split into subdomains, NTD (24–289) and RBD (334–528), for rigid-body refinement. Simulated annealing was performed initially, including a local grid search and ADP refinement, using secondary structure restraints. We noticed that the original 6XKL model lacked some loops in RBD and NTD, which were replaced by the RBD from 7KGJ and the NTD from 7B62 (58) with all loops. For the model of the “1-up” form of S-6P, the CC was 0.84/0.78 (volume/peaks), with three Sb45 domains bound to three RBDs. However, the CCs for three Sb45-X, Sb45-Y, and Sb45-Z are 0.51, 0.49, and 0.58, respectively, which indicates that Sb45 does not fully bind to S-6P. For the “2-up” form of S-6P, we first generated the model by superimposing the A chain of the “1-up” form of S-6P onto the B chain and replaced the B chain for the real-space refinement. The resulting model was with an overall CC of 0.83/0.76 (volume/peaks), but with only two Sb45 domains, one Sb45-X binds to the A chain (up RBD) and one Sb45-Z binds to the C chain (down RBD) with CCs of 0.44 and 0.68, respectively. These two models are deposited in the PDB as 7N0G and 7N0H. Data processing, refinement statistics, and model validation are listed in Table 2.

## Data availability

All data are included in the article or the supplemental material. The X-ray structure factors and coordinates are deposited at the Protein Data Bank (www.pdb.org) under accession numbers 7MFU, 7KGK, 7KGJ, 7KLW, and 7MFV for Sb14–RBD–Sb68, Sb16–RBD, Sb45–RBD, Sb45–RBD–Sb68, and Sb16, respectively. The cryo-EM maps of SB45+S-6P (1-up, 2-down) and Sb45+S-6P (2-up, 1-down) have been deposited in the Electron Microscopy Data Bank under accession numbers EMD-24105 and EMD-24106, respectively, and their respective models under 7N0G and 7N0H.

## Supporting information

This article contains supporting information.

## Conflict of interest

The authors declare that they have no conflicts of interest with the contents of this article.
